# Editorial: Emerging trends in headache

**DOI:** 10.3389/fpain.2023.1288707

**Published:** 2023-10-19

**Authors:** Luca Giani, Pınar Yalinay Dikmen

**Affiliations:** ^1^ Department of Neurorehabilitation, Istituti Clinici Scientifici Maugeri IRCCS, Milan, Italy; ^2^ School of Medicine, Department of Neurology, Maslak Acıbadem Hospital, Acıbadem University, Istanbul, Türkiye

**Keywords:** migraine, genetic, headache, fMRI, gender medicine, COVID-19

**Editorial on the Research Topic** Emerging trends in headache

Headaches are a common ailment that afflicts individuals across the globe, transcending age, gender, and geographic boundaries. They can be debilitating, impacting the quality of life and productivity of those who suffer from them (1).

In recent years, the scientific community has made significant strides in understanding the complexities of headaches, particularly migraine, and has explored novel approaches to their diagnosis, treatment, and management. The collection of papers presented in this Research Topic, “Emerging Trends in Headache”, showcases some of the latest advancements in this field, shedding light on various aspects of headaches and their implications for healthcare. They represent a diverse range of research, from basic science to clinical real-world experience.

Migraine is one of the most frequent and disabling diseases worldwide (1). Its etiopathogenesis is complex and involves a combination of factors. Multiple genetic variants contribute to an individual's susceptibility risk, with each variant typically exerting a modest influence (2). Genes interact with an intricate web of influences encompassing endocrine and metabolic factors, comorbidities, environmental influences, life events, and behavioral choices. These dynamic forces converge to determine the presentation of the disease and shape its manifestation. Migraine is associated with obesity and diabetes mellitus (3), depression, anxiety, post-traumatic stress disorder, and sleep disorders (4). Increasing migraine headache frequency was associated with gastric ulcers, diabetes, anxiety, depression, insomnia, asthma and allergies (5). Recent studies highlight a link between the gut-brain-immune (GBI) axis and migraine (6). These associations imply bidirectional causality, shared genetic factors, and/or common pathophysiological mechanisms (7).

Understanding these determinants, their interactions, and the biological mechanisms thereof, is a multidisciplinary endeavor with the potential to uncover innovative and better management strategies.

Fitzgerald et al.'s paper, “Sex Differences in Migraine: A Twin Study”, explores the intriguing dimension of gender disparities in migraine. Migraine is known to affect women disproportionately, and this twin study delves into the genetic and environmental factors contributing to this phenomenon. The study found that migraine is equally heritable in females and males, but that there may be subtle differences in the genes underlying migraine across the sexes. The study also found that females with a male co-twin have an increased risk of migraine, suggesting that masculinization of the prenatal environment may increase migraine risk for females. The authors suggest a potential prenatal neuroendocrine factor in the development of migraine. Migraine and migraine related syndromes can indeed begin in the early infancy (8). Migraine is a multifactorial condition, with its features probably arising from gene–gene and gene–environment interactions. It is stimulating to think that such environmental exposure can act as early as the intrauterine life. Throughout the life of a subject, these interactions shape the development of the brain and influence the connectivity between brain areas through plastic remodeling (9), with significant consequences on health. For example, Gecse et al. found that there are sex differences in the functional connectivity of the periaqueductal gray matter (PAG), a region of the brain that is involved in pain processing. The study suggests that these sex differences contribute to the different clinical presentations of migraine in females and males.

Calcitonin gene related peptide (CGRP) is a peptide that participates in the transmission of pain signals. CGRP inhibitors are a new class of drugs that are effective in preventing migraine attacks. As new drugs are being introduced on the market, important questions arise about real world experiences, intentional and unintentional effects of combinations and interactions. The paper by Shah et al. gives a review of the current evidence about the effects of combining different CGRP inhibitors in the same patient.

The COVID-19 pandemic has brought forth an array of health challenges, including a surge in post-vaccination side effects (10). The paper by Ceccardi et al. investigates the incidence of headache after COVID-19 vaccination. The study found that headache is a common side effect of vaccination, but that it is more likely to occur in people who have a history of migraine. The study also found that headache after vaccination is usually benign, mild and resolves on its own within a few days. This article provides some of the first insights into the incidence and management of headache in this population and offers some clinical hints for distinguishing the rare severe forms.

The articles comprising this Research Topic illuminate significant advancements in headache research, yielding fresh insights into its etiology and mechanisms while propelling the development of novel, efficacious therapies.

Within these papers, emerging trends in headache research are discernible. These trends encompass the intricate interplay of genetic and environmental factors underpinning migraine. They involve the application of neuroimaging techniques to scrutinize its cerebral mechanisms. Exciting new preventative and treatment modalities are entering clinical practice, warranting optimal management strategies. Moreover, the COVID-19 pandemic has introduced novel challenges into both our daily lives and clinical endeavors.

As we contemplate these four papers, it becomes apparent that the headache research landscape is in a state of rapid evolution. These studies collectively underscore the significance of incorporating gender-specific variables into migraine investigations, probing innovative neurobiological pathways, and exploring inventive therapeutic interventions. Furthermore, they underscore the ongoing necessity for vigilant health outcome monitoring during the ongoing global vaccination campaigns.

Headaches persist as a multifaceted issue, and it is heartening to observe interdisciplinary cooperation among researchers in unraveling their complexities. This collection signifies a juncture where genetics, neurobiology, pharmacology, and epidemiology converge, ushering in new avenues for headache research and treatment. Our aspiration is that these nascent trends will kindle further inquiries, culminating in enhanced management and relief for those afflicted by headaches.

We anticipate that this Research Topic will captivate the interests of researchers, clinicians, and patients alike. We believe that the body of work presented herein constitutes a substantial addition to the headache research domain.

We would like to thank all the authors who contributed to this Research Topic. Their work is helping to improve our understanding of headache and to develop new treatments for this common and disabling condition.
